# Religious Involvement and Mental Disorders in Mainland China

**DOI:** 10.1371/journal.pone.0128800

**Published:** 2015-06-01

**Authors:** Zhizhong Wang, Harold G. Koenig, Yuhong Zhang, Wanrui Ma, Yueqin Huang

**Affiliations:** 1 Department of Epidemiology and Statistics, School of Public Health, Ningxia Medical University, Yinchuan, China; 2 Department of Psychiatry and Behavioral Sciences, and Department of Medicine, Duke University Medical Center, Durham, North Carolina, United States of America; 3 Department of Medicine, King Abdulaziz University (KAU), Jeddah, Saudi Arabia; 4 Comprehensive Department, General Hospital of Ningxia Medical University, Yinchuan, China; 5 Peking University Sixth Hospital, Peking University Institute of Mental Health, Key Laboratory of Mental Health, Ministry of Health (Peking University), Beijing, China; Institute of Psychiatry, UNITED KINGDOM

## Abstract

**Purpose:**

The present study aims to examine the association between religious involvement and mental disorder (anxiety disorder, mood disorder, alcohol use disorder) in a general Chinese population, and explore connections between religious belief and mental disorders in the Hui and Han ethnic groups.

**Method:**

Data were examined from a representative sample of 2,770 community-dwelling adults in the province of Ningxia located in western China. Self-reported religious attendance and the importance of religious in daily life were measured. The WHO Composite International Diagnostic Interview was used to diagnose mental disorders.

**Results:**

In the overall sample, the importance of religious affiliation was positively associated with mental disorders (especially anxiety) (p<0.01). No association was found between any religious characteristic and mood disorders or alcohol use disorders. With regard to analyses within different ethnic groups, religious affiliation was positively associated with mental disorder in Han ethnicity (p<0.01), but not in Hui ethnicity. When stratified by age and ethnic group, religious affiliation was associated positively with mental disorder in younger Han (p<0.01); whereas high religiosity was associated positively with mental disorder in older Hui (p<0.05). Among older Hui, however, religious affiliation was inversely associated with mood disorder (p<0.05).

**Conclusions:**

In contrast to most previous studies in Western populations, religious involvement is less likely to be inversely related to mental disorder in Mainland China, although this association varies by age and ethnic group.

## Introduction

The relationship between religiosity and mental health is receiving more and more attention in the research literature [1,2]. Koenig *et al* reviewed studies published prior to 2010 on the association between religion and mental health, finding that 67% of the methodologically most rigorous studies reported an inverse association between religious involvement and depression, and 90% of studies reported an inverse relationship with alcohol use disorders[3]. Another review found that 100% of studies published between 1990 and 2010 found an inverse relationship with neurosis[1]. Most recently, a 14 years follow-up study of a random sample of 12,583 adults in Canada found that attending religious services monthly predicted a 22% reduced risk of developing major depression [4], and a ten-year prospective study showed that a higher self-reported rating of the importance of religion or spirituality decreased the risk of major depression by 90% in adults at high risk for depression[5], and those at risk who indicated very high importance of religion or spirituality also had thicker brain regions on structural MRI in areas that tended to be atrophied in those at high risk but lower importance of religion [6].

Furthermore, many researchers have begun to integrate the religious beliefs of patients into psychotherapy for the treatment of mental disorder [7–10]. One review reported that 71% of studies found that religious or spiritual interventions reduced anxiety[3].

Most of these studies, however, have focused on Christian populations, and have typically been conducted in countries that are generally religious and well-developed economically. Is the connection of religion and mental health robust across cultures? Many scholars have answered this question in the affirmatively. Koenig and colleagues reviewed studies that examined relationships over the past 100 years between religion and health (over 70% involving religion and mental health) throughout the world, found in both Western and Middle-Eastern countries a positive link between religious involvement and mental health [1,11]. Little research, however, has been done in non-Christian cultures like the one that exists in China.

Also, Yeager *et al* has noted that studies in literature may be influenced by publication bias in that journals tend to publish studies that report significant findings, but tend not to publish studies that find no association [12].

China is believed to be less religious than many Western countries [13]. Despite this, China has a diverse religious population that consists of Buddhists, Taoists, Muslims, Jews, Christians and a variety of other Chinese religions[14]. As a result of the increased desire on the part of Chinese people for religious freedom, and current social and economic changes that are occurring in China, there has been a rapid increase in the percentage who claim some type of religious practice (from 7.0% in 2001 to 23.9% in 2007) [15]. According to the Pew Research Center, over 40% of Chinese are affiliated with at least one religion. Globally this means that more than seven-in-ten (73%) of the world’s folk religionists, and half (50%) of the world’s Buddhists live in China [16].

Few studies have focused on the association between religious involvement and mental disorders in mainland China. Nevertheless, a small amount of research has emerged over the past few years on religious involvement and psychological well-being[17], depressive symptoms[18,19], suicidal ideation[19,20], cognitive impairment[21,22], and mortality [23,24] in Mainland China.

The present study takes place in the Ningxia province of China, where over one-third of the population is Hui ethnicity, which is a minority group in China that is largely descended from those who came to China from Saudi Arabia seeking work; consequently, this ethnic group is composed almost entirely of Muslims [25, 26]. Most of the population of China and the rest of the population in the Ningxia province is of Han ethnicity, which if affiliated with any religious group, is usually affiliated with Chinese religions such as Buddhist, Doaism, Confucianism, and others[14]. Few studies compare these two ethnic groups in terms of their mental health or their religious involvement.

To address gaps in the literature, the present study examined the association between religious involvement and mental disorders in a large, representative sample of community-dwelling adults in Mainland China. The objectives of this study were to (1) examine the association between religious involvement and mental disorders (anxiety disorders, mood disorders, alcohol use disorders, any mental disorders); (2) examine these relationships controlling for socio-demographic and physical health factors; (3) explore the connections between religious belief and mental disorder in Chinese Hui Muslims compared to those Chinese Han; and (4) examine interactions with age and ethnic group.

## Methods

### Participants

Participants were from a population-based epidemiological study of mental disorders in the province of Ningxia, located in western China, where the Hui ethnicity makes up 35% of the total population (6.4 million)[27]. Inclusion criteria were: aged 18 years or older and in residence for at least six months at their current address. Exclusion criteria were: unconsciousness caused by brain injury, brain tumor and/or craniotomy or dementia; being in the acute phase of a cerebrovascular accident; experiencing a severe illness that prevented communication; having any obvious cognitive disabilities; or currently suffering from deafness, aphasia or other language barriers.

### Procedure

Participants were selected in three stages. First, 62 primary sample units (PSU) were selected from 2,209 villages and 393 neighborhood communities using a probability proportionate to size (PPS)[28] method. Second, depending on the total number of households in the selected PSU, 60 to 210 households were identified from each PSU using a systematic sampling method, resulting in a total of 6,890 households being selected. Third, interviewers visited each household and used a Kish selection table[29]to randomly select one eligible participant from each household. Interviewers were unable to reach the household member selected in 414 cases, resulting in a total sample of 6,476 participants who were approached to conduct a face-to-face interview from July 2011 to January 2013. Of those, 5,810 participants (89.7%) completed the interview. The present study consisted of 2,770 participants who completed the Part II interviews described below.

Face-to-face, computer assisted personal interviews (CAPI) [30] were carried out by lay interviewers from Ningxia Medical University. Interviewers were trained in a 7-day training session by our research team. The training covered general interviewing techniques, review of the questionnaire, post-interview editing and in- and out-of-classroom exercises for interviewers. 90 trainees passed the final test and were selected to be interviewers.

The interview schedule was divided into two parts. Part I, which was administered to all respondents, included the core WHO-CIDI diagnostic interview for mental disorders. Part II included assessments of risk factors, services sought, religious involvement, and assessment of additional disorders that were either of secondary importance or were too time consuming to assess in the full sample [31]. Selection of subjects to complete Part II was controlled by the CAPI program, which divided respondents into three groups based on their Part I responses. First, all respondents who (1) met lifetime criteria for at least one mental disorder assessed in Part I, (2) met sub-threshold lifetime criteria for a mental disorder and sought treatment for it at some time in their life, or (3) either ever made a plan to commit suicide or attempted suicide, were selected to complete Part II of the evaluation. Second, a probability sample was selected of 59% of respondents who did not meet criteria for membership in the first group, but gave responses in Part I indicating that they (1) ever met subthreshold criteria for Part I disorders, (2) ever sought treatment for any emotional or substance abuse problem, (3) ever had suicidal ideation, or (4) used psychotropic medications in the past 12 months to treat emotional problems. Third, a 25% random sample of respondents without mental disorders or emotional problems was selected to receive the Part II evaluation [31].

### Ethics statement

In order to eliminate the influence that the stigma towards mental illness might have and encourage participants to report their mental symptoms, the survey was designed as anonymous. The potential risks and benefits of the survey were described by the interviewer and the participants were asked to provide their consent by checking a box on computer screen with the response (1 = I agree to participate in the study; 5 = I do not agree to participate in the study). If the response was “I do not agree”, the CAPI program was immediately terminated automatically. The consent document was recorded as one of the variables in the dataset file by computer program. The study was approved by the Institutional Review Board of the Ningxia Medical University.

### Measurement

#### Dependent variables

Twelve-month prevalence of mental disorders (Anxiety disorders include agoraphobia, generalized anxiety disorder, obsessive-compulsive disorder, panic disorder, social phobia, specific phobia, and neurasthenia; Mood disorders include unipolar depressive disorder and bipolar disorder; Alcohol use disorders) were assessed. The WHO Composite International Diagnostic Interview (WHO-CIDI) [32] was used to diagnose mental disorders according to the International Classification of Disease 10th Edition (ICD-10) diagnostic criteria. The Training and Recourse Center of CIDI in Beijing provided the Chinese version of the WHO-CIDI-CAPI and training program. Culture adaptation and modification research found it was a good instruction in validation [33]. High concordance was found between the clinical evaluation for mental disorder and the Chinese version CIDI diagnoses [34], and consequently has been widely used in epidemiological studies in China[35,36].

#### Independent variables

As a section of full CIDI, religious participation and the importance of religion were measured by asking: “How often do you usually attend religious activities?” (more than once a week, about once a week, one to three times a month, less than once a month, never) and “In general, how important are religious or spiritual beliefs in your daily life?” (very important, somewhat, not very, or not at all important). High religiosity was defined as both (1) attending religious activities at least 2–3 times per month and (2) religious or spiritual beliefs being very important in daily life. Religious affiliation was determined by asking “What is your religion?”

Socio-demographic information was collected using the demographic section of the CIDI. Demographic characteristics included age, gender, education, marital status (married vs. unmarried), residence (rural vs. urban), ethnicity (Han as majority vs. Hui as minority in China), experience of migration from other areas of China (yes vs. no), and geographical region (developed vs. undeveloped).

Physical health variables included overall self-reported physical health (good vs. poor), self-reported chronic body pain (yes vs. no), type II diabetes (yes vs. no), and hypertension (yes vs. no).

### Statistical Analyses

Analyses were performed using the Statistical Analysis System (SAS) 8.2 software (SAS Institute Inc. Cary, NC, USA). Differences in socio-demographical characteristics between Hui and Han ethnicities, and associations between participant characteristics and mental disorders, were examined using one-way-analysis of variance for continuous variables, and the chi-square statistic for categorical variables, Wilcoxon-Mann-Whitney test for religious attendance and importance. No weighting program applied during the comparison analysis. The data were not weighted for comparison analysis.

Three separate unconditional logistic regression models were used to examine associations between religious involvement and mental disorders. In summary, model 1 included the religious involvement variable; model 2 included the religious variable and socio-demographic characteristics; and physical health variables were added in model 3. Analyses were stratified by ethnicity and age (under age 50 and age 50 or over) for model 3. Religious affiliation was recorded as a binary variable (with vs. without a religious affiliation).

Odds ratio with 95% confidence interval were calculated for all models. Given the exploratory nature of these analyses, statistical significance level was set at 0.05, without corrections for multiple comparisons.

## Results

### Sample Characteristics

Demographic, health, and religious characteristics of the sample are presented in Table 1. The final sample consisted of 2,770 participants (39.9% Muslim, 8.9% Chinese religions, 1.9% Christians, 49.3% no religious affiliation). The average age was 44.3 years (SD 15.2) with a range from 18 to 89. The average education level was 5.9 years (SD 4.9) with a range from 0 to 17. The majority were female (62.4%), and from developing areas (61.9%) where people live on less income (average Gross Domestic Product less than $1,987 per year in 2012). Nearly a quarter of the sample (22.0%) met ICD-10 criteria for one or more mental disorder. Most common was anxiety disorders (15.8%), less common was mood disorder (4.0%), and the least common was alcohol use disorder (1.0%). Compared to participants of Han ethnicity, Hui were younger and had less education(*P*<0.001), and also reported poorer physical health, higher physical pain, and were more likely exposed to anxiety disorder or any mental disorders (p<0.001).

**Table 1:** Characteristics of the sample.

	Total	Hui	Han ^#^
	N = 2,770	N = 1,150	N = 1,620
**Demographics**			
Age, years, mean(sd)	41.4(14.6)	41.5 (14.4)	46.3 (15.3) ^‡^
Education, years, mean(sd)	5.9(4.9)	4.6 (4.7)	6.8 (4.9) ^‡^
Gender, male,% (n)	37.6(1,042)	37.6 (433)	37.6 (609)
Marriage, married, % (n)	88.1(2,441)	89.5 (1,029)	87.2 (1412)
Region, developing, % (n)	61.9(1,715)	51.3 (590)	69.4 (1,125) ^‡^
Migrant, yes, % (n)	30.9(857)	45.5 (523)	20.6 (334) ^‡^
Urban/rural, rural, % (n)	73.9(2,048)	84.3 (969)	66.6 (1,079) ^‡^
**Physical health**			
Overall physical health, poor	41.6(1,152)	38.7 (445)	43.6 (707) ^†^
Type II diabetes, yes	3.7(103)	3.0 (35)	4.2 (68)
Hypertension, yes	14.3(396)	11.8 (136)	16.1 (260) ^†^
Physical pain, yes	50.9(1,411)	56.8 (653)	46.8 (758) ^‡^
**Mental disorders**			
Anxiety disorders, yes	15.8 (439)	20.3 (233)	12.7 (206) ^‡^
Mood disorders, yes	4.0 (110)	4.3 (50)	3.7 (60)
Alcohol use disorders, yes	1.0 (29)	0.6 (7)	1.4 (22)
Any mental disorders, yes	22.0 (609)	26.7 (308)	18.6 (301) ^‡^
**Religious involvement**			
Religion participation, median (Q_1-3_)	1 (1, 2)	3 (2, 5)	1 (1, 1) ^‡^
Importance of religion, median (Q_1-3_)	3 (1, 4)	4 (4, 4)	2 (1, 3) ^‡^
High religiosity, % (n)	20.1 (556)	45.6 (525)	1.9 (31) ^‡^
**Religious affiliation**			
Buddhist/Taoist, % (n)	8.9 (246)	0.1 (1)	15.1 (245) ^‡^
Muslim,% (n)	39.8 (1,103)	93.0 (1,071)	2.0 (33)
Christian/Catholic, % (n)	2.0 (54)	1.2 (17)	2.3 (37)
No affiliation, % (n)	49.3 (1,367)	4.4 (62)	80.6 (1,305)

^†^: P<0.01

^‡^: P<0.001.

^**#**^ compared with the Hui ethnic group.

Q_1-3_: quartile.

Most Hui were Muslim (93.0%), with significantly higher religious participation and greater importance of religion compared to those of Han ethnicity (p<0.001). In the entire sample, 20.1% met criteria for high religiosity, with 45.6% of Hui doing so compared to 1.9% of Han. Younger participants (under age 50) were less likely to engage in religious participation compared to older participants (with 2.9 vs. 3.4 for Hui, p<0.001; 1.1 vs. 1.2 for Han, p<0.001). A smaller proportion of younger participants also met criteria for high religiosity compared to older participants in both Hui (42.3% vs. 54.4%, p<0.001) and Han (1.4% vs. 2.7%, p = 0.05).

### Bivariate Associations

Table 2 displays associations between participant characteristics and mental disorders. Participants with lower education, living in developing regions, migrants from other provinces in China, and those from rural areas were more likely to have a mental disorder. Men were at higher risk than women for alcohol use disorders (p<0.001), whereas women were at higher risk for anxiety disorders (p<0.001). Poorer overall physical health and reports of physical pain were associated with anxiety disorder and any mental disorder (p<0.001).

**Table 2:** Bivariate associations between participant characteristics and mental disorders (N = 2,770 for all comparisons)

	Any MD	Anxiety	Mood	Alcoholism
	*Chi* ^*2*^ */ F value*	*Chi* ^*2*^ */ F value*	*Chi* ^*2*^ */ F value*	*Chi* ^*2*^ */ F value*
**Demographics**				
Age, years (younger)	0.60	0.29	0.06	4.4*
Education, years (lower) ^a^	13.01^‡^	24.43^‡^	3.89*	8.9 ^†^
Gender (female) ^b^	2.93	20.48***	0.46	38.44^‡^
Marriage (unmarried)	0.64	0.01	8.92 ^†^	0.06
Region (developing)	15.04^‡^	13.90^‡^	4.95*	0.61
Migrant (yes)	16.22^‡^	18.46^‡^	8.64 ^†^	4.03*
Urban/rural (rural)	7.80 ^†^	10.56^†^	3.69	3.56
**Physical health**				
Overall health (poor)	38.05^‡^	45.03^‡^	9.66 ^†^	1.23
Type II diabetes (yes)	2.59	0.40	2.52	0.01
Hypertension (yes)	0.06	0.60	0.57	1.30
Any physical pains (yes)	57.71^‡^	66.54^‡^	3.04	0.21
**Religious involvement**				
Religious affiliation (yes)	31.89^‡^	38.52^‡^	0.02	1.00
Religion participation (frequent)	21.09^‡^	20.89^‡^	1.61	1.01
Importance of religion (very) ^c^	26.74^‡^	31.16^‡^	0.97	3.84*
High religiosity (yes)	16.78^‡^	12.19^‡^	2.82	0.14

() characteristic associated with higher risk of mental disorder.

^a^ alcohol use disorder is more common in those with higher education.

^b^ alcohol use disorder is more common in male.

^c^ alcohol use disorder is more frequent among those with less importance.

Any MD: any mental disorder.

*: P<0.05

^†^: P<0.01

^‡^: P<0.001.

Greater religious involvement was also associated with a higher risk of anxiety disorder and any mental disorder, but was not associated with mood disorder. The importance of religion was inversely associated with alcohol use disorders (p<0.05).

### Multivariate Analyses

In model 1, a positive association was found between religious affiliation, any mental disorder, and anxiety disorder in particular (p<.001) (Table 3). Same was true between high religiosity and any mental disorders, and anxiety disorder (P<.001). In model 2, the association of religious affiliation and any mental disorder (p<0.01), and anxiety disorder (p<0.01) persisted after controlling for demographic variables. No association, however, was found between mental disorder and high religiosity. Model 3, controlling for both demographic and physical health variables, indicated similar results. No significant association was found between religious involvement and mood disorder or alcohol use disorder in any of the three models.

**Table 3:** The association between religious involvement and any mental disorders (N = 2,770).

	Model 1	Model 2	Model 3
	*OR(95%CI)*	*OR(95%CI)*	*OR(95%CI)*
**Any Mental Disorders**			
Religious affiliation	1.6 (1.4–2.0) ^‡^	1.4 (1.1–1.8) ^†^	1.4 (1.0–1.8) *
Model R-square	0.01	0.02	0.04
High religiosity	1.5 (1.2–1.9) ^‡^	1.2 (0.9–1.5)	1.2 (0.9–1.6)
Model R-square	0.00	0.02	0.04
**Anxiety Disorders**			
Religious affiliation	1.9 (1.5–2.3) ^‡^	1.6 (1.2–2.2) ^†^	1.6 (1.2–2.2) ^†^
Model R-square	0.01	0.03	0.05
High religiosity	1.5 (1.2–1.9) ^‡^	1.1 (0.8–1.4)	1.1 (0.8–1.5)
Model R-square	0.00	0.02	0.05
**Mood Disorders**			
Religious affiliation	0.9 (0.6–1.4)	0.6 (0.3–1.2)	0.6 (0.3–1.2)
Model R-square	0.00	0.01	0.01
High religiosity	1.4 (0.9–2.2)	1.4 (0.8–2.5)	1.4 (0.8–2.5)
Model R-square	0.00	0.00	0.01
**Alcohol Use Disorders**			
Religious affiliation	0.6 (0.3–1.4)	1.2 (0.4–3.3)	1.3 (0.4–3.4)
Model R-square	0.00	0.02	0.02
High religiosity	0.8 (0.3–2.1)	1.9 (0.4–8.6)	1.9 (0.4–8.9)
Model R-square	0.00	0.01	0.02

Model l = religious variable; Model 2 = Model l + demographics; Model 3 = Model 2 + physical health.

OR: Odds ratio, 95%CI: 95% confidence interval.

*: P<0.05

^†^: P<0.01

^‡^: P<0.001.

### Stratification by Ethnic Group

Multivariate models revealed inconsistent associations between religious involvement and mental disorder when analyses were stratified by ethnic group (Hui vs. Han). In those of Han ethnicity, religious affiliation was positively associated with having a mental disorder (any) (p<0.01), and having an anxiety disorder in particular (p<0.01), although this was not present for those of Hui ethnicity (Table 4). No association was found between high religiosity and either mood disorder in both the Hui and the Han. Due to the small positive sample size of alcohol use disorders, we have not performed it at the stratification analysis.

**Table 4:** Unconditional Logistic regression analyses examining relationships between religiosity and mental disorders stratified by ethnic.

	Hui (N = 1,150)	Han (N = 1,620)
	*OR(95%CI)*	*OR(95%CI)*
**Any Mental Disorders**		
Religious affiliation	1.0 (0.5–1.9)	1.5 (1.1–2.0) ^†^
Model R-square	0.03	0.04
High religiosity	1.3 (0.9–1.7)	1.0 (0.4–2.7)
Model R-square	0.03	0.03
**Anxiety Disorders**		
Religious affiliation	1.1 (0.5–2.3)	1.7 (1.2–2.5) ^†^
Model R-square	0.04	0.05
High religiosity	1.1 (0.8–1.5)	0.9 (0.3–2.7)
Model R-square	0.04	0.05
**Mood Disorders**		
Religious affiliation	0.4 (0.1–1.1)	0.7 (0.3–1.6)
Model R-square	0.02	0.01
High religiosity	1.3 (0.7–2.4)	1.52 (0.3–6.7)
Model R-square	0.01	0.01

Model = religious variable + demographics + physical health.

OR: Odds ratio, 95%CI: 95% confidence interval.

^†^: P<0.01.

### Stratification by Ethnic Group and Age

Analyses were then stratified by ethnic group and age (Table 5). Religious affiliation was associated with a greater risk of anxiety disorder and any mental disorder in younger Han (p<0.01). Overall religiosity (high) was positively associated with anxiety disorder and any mental disorder in older Hui (p<0.05). In contrast, however, there was an inverse association between religious affiliation and mood disorder in older Hui (p<0.05).

**Table 5:** The association between religious involvements and mental disorders stratified by age.

	Hui (N = 1150)		Han (N = 1620)	
	Age <50	Age > = 50	Age <50	Age > = 50
	N = 869	N = 281	N = 1,011	N = 609
	*OR(95%CI)*	*OR(95%CI)*	*OR(95%CI)*	*OR(95%CI)*
**Any Mental Disorders**				
Religious affiliation	0.9 (0.4–1.9)	1.1 (0.3–3.8)	1.7 (1.1–2.5) ^†^	1.2 (0.7–2.0)
Model R-square	0.03	0.07	0.05	0.04
High religiosity	1.2 (0.8–1.6)	1.8 (1.0–3.3) *	1.6 (0.4–5.4)	0.6 (0.1–2.7)
Model R-square	0.03	0.08	0.05	0.04
**Anxiety Disorders**				
Religious affiliation	0.9 (0.4–2.0)	2.0 (0.4–9.5)	2.0 (1.3–3.1) ^†^	1.4 (0.8–2.5)
Model R-square	0.03	0.08	0.07	0.04
High religiosity	0.9 (0.6–1.4)	2.0 (1.0–3.8) *	0.9 (0.2–4.3)	0.9 (0.2–4.3)
Model R-square	0.03	0.08	0.06	0.04
**Mood Disorders**				
Religious affiliation	0.5 (0.1–2.6)	0.1 (0.0–0.8) *	1.0 (0.4–2.3)	0.5 (0.1–1.9)
Model R-square	0.03	0.05	0.02	0.03
High religiosity	1.5 (0.7–3.2)	0.8 (0.2–2.8)	4.6 (0.9–22.6)	—
Model R-square	0.03	0.04	0.02	0.03

Model = religious variable + demographics+physical health.

OR: Odds ratio, 95%CI: 95% confidence interval.

—: no proper OR estimated due to the few positive high religiosity cases in the group.

*: P<0.05

^†^: P<0.01.

## Discussion

The present study examined the relationship between religious involvement and mental disorders among community dwelling adults in China. Only 1.9% Chinese Han attend religious service frequently and indicate that religion is very important in their lives, compared with studies in the Western countries (69% British[37], more than 50% of Americans say religion is very important in daily life and attend religious services at least monthly [38,39]). These findings are consistent with those from the National Latino and Asian American Study (NLAAS) that reported 61% of Chinese Americans never attend religious services [40].

In the present study, those of Hui ethnicity scored higher on religiosity than those of Han ethnicity due to different religious affiliations and different ways that they express religious practice. Buddhism is the most popular religion among the Han[41], and believers usually practice their religion in the privacy of their homes or in ancestral halls. Some of Buddhist temples are built in the mountains or by the riverside, far away from the cities. This makes it more difficult for believers to worship together at the temple. Most Hui are Muslims who attend religious services at the local mosque, where the imam gives a sermon about practical issues in life every Friday, and worship and prayer takes place five times a day either in the mosque or at home[11,42].

Surprisingly, those who have religious affiliation in our study had the higher risk of mental disorder. What are some possible explanations for this finding, which contrasts with much of the research from non-Asian countries? One of the possible explanation is that religious involvement in China generates or causes mental health problems, especially anxiety over religious doctrines related to guilt and punishment for sins during this life and the next life, causing neurosis (as Freud and others have argued)[43]. Religion may promote psychosocial strains that result from struggles that people have when trying to live up to the high standards set by their faith traditions[44]. People may also feel abandoned or punished by God when experiencing difficult life situations, resulting in higher mental distress [45]. One study reported that religious and moral issues among Muslims generated stress among women leading to higher mental distress [46].

There is, however, another possibility. Many people are known to turn to religion for comfort and strength during times of adversity[47]. For example, Greenberg evaluated the religious background of newly orthodox Jews, and concluded initially that their religious conversion had precipitated their mental problems. However, the majority of participants had a history of mental health problems prior to their religious change, and that change brought many relief from their suffering for many years until their earlier mental health problems later re-emerged [48,49]. In one study of Chinese Christians, researchers found that the median age-of-onset of mental disorder was 25years old [50], earlier than median age of reported religious change (32.7 years old) [51]. Along a similar vein, Schnittker’s examination of religious salience using a nationally representative sample of Americans indicated that those with very low or very high levels of religious salience were more likely to have depressive symptoms [52].

Furthermore, religious awakening often occurs under conditions of social upheaval. Chinese society has experienced great changes and a dramatic cultural revolution, resulting in a large proportion of the population suffering severe economic hardship and competition for resources, with an increased prevalence of mental disorders[53], similar to what happened 100 years ago[54]. Many of those in the “high distress” group were attracted to religious belief because of their dissatisfaction with secular life that they believed caused mental distress and negative life events [55].

Interestingly, the present study found that high religiosity was associated with greater anxiety, but less mood disorder in elder Hui participants. This finding seems contradictory since mood disorder and anxiety disorders often go together and have a heavy overlap in symptoms, and so this finding is difficult to explain. Perhaps older Hui were more likely to turn to religion for relaxation and a sense of peace in order to control their anxiety, creating a positive relationship between religiousness and anxiety. Scholars have proposed that a primary psychological function of religion is to comfort the self [56]. Elderly persons, in particular when they encounter difficulties and helplessness, may find in religion reasons to explain and give purpose to their physical or mental suffering, as well as offering to them the promise of comfort in the afterlife [57]. In contrast, we know that depression may prevent people from attending religious services because it causes a reduction in energy, reduction in motivation, and increase in social withdrawal, which may help to explain the inverse relationship between religiosity and mood disorders in older adults. Furthermore, the findings regarding the relationship between religious involvement and mental health vary depending on the aspect of religion measured and the mental health outcome examined[58].

The present study has several limitations. First, is the cross-sectional design, making it impossible to determine whether higher religiosity led to mental disorder, or whether mental disorder caused people turn to religion for comfort or solace. Second, as with any study based on subjective self-report, there is the potential for recall bias which could have influenced the accuracy of the data reported. Third, only 29 participants (1.0%) had an alcohol use disorder, and not a single participant in the elderly Hui ethnicity group met that criteria. Given the small size, this study had little power to detect associations between religious involvement and alcohol disorder. Power was also an issue for examining associations between high religiosity and mental disorder in the Han ethnic group, where only 31 (1.9%) participants met criteria for high religiosity and only 13 (1.4%) of those in the younger group met those criteria. Finally, the sample selected for this study was from only one province in China, so generalizing these results with other areas of China must be done with caution.

## Conclusions

The present study’s findings are important for several reasons. Foremost, this is the first study to our knowledge to focus on the association between religious involvement and mental disorder using a community-based sample in Mainland China. Second, this study examined the association between religious involvement and mental disorder in different ethnic groups with distinctively different religious affiliations (the mainstream Chinese religions in those of Han ethnicity versus the Muslim religion in those of Hui ethnicity), which groups are often overlooked groups in previous studies of religion and mental health. Finally, this study provides baseline data for further future research in this field under the religious awakening era in China.
